# *N*′-[(*E*)-3-Bromo­benzyl­idene]pyrazine-2-carbohydrazide

**DOI:** 10.1107/S1600536813027426

**Published:** 2013-10-12

**Authors:** Mushtaq Ahmad, Shahid Hameed, M. Nawaz Tahir, Muhammad Anwar, Muhammad Israr

**Affiliations:** a Department of Chemistry, Quaid-i-Azam University, Islamabad, Pakistan; b Medicinal Botanic Centre, PCSIR Laboratories Complex, Peshawar, Pakistan; c University of Sargodha, Department of Physics, Sargodha, Pakistan; d Department of Chemistry, Kohat University of Science and Technology, Kohat, Pakistan

## Abstract

In the title compound, C_12_H_9_BrN_4_O, the dihedral angle between the aromatic rings is 12.16 (12)°. An intra­molecular N—H⋯N hydrogen bond closes an *S*(5) ring. In the crystal, C—H⋯O hydrogen bonds link the mol­ecules into *C*(6) chains propagating in [010]. Very weak aromatic π–π stacking [centroid–centroid separations = 3.9189 (15) and 3.9357 (15) Å] is also observed.

## Related literature

For related structures, see: Hameed *et al.* (2013*a* ▶,*b* ▶).
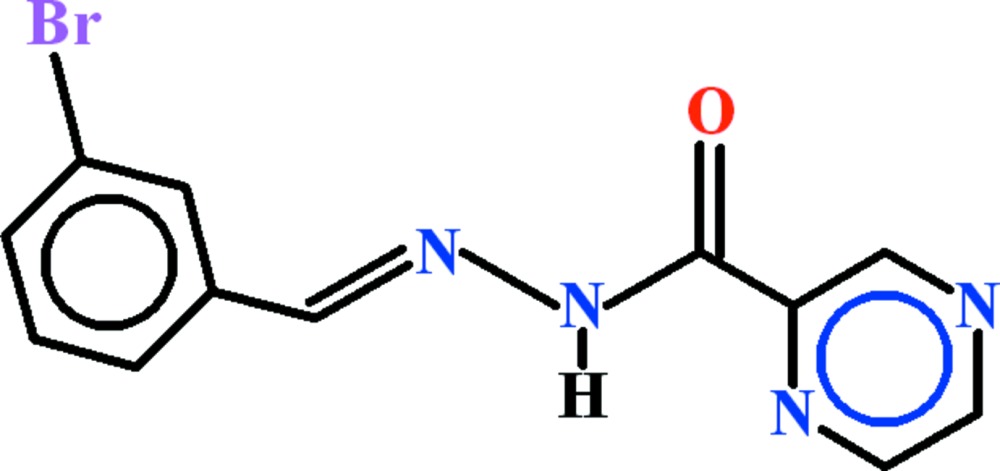


## Experimental

### 

#### Crystal data

C_12_H_9_BrN_4_O
*M* *_r_* = 305.14
Monoclinic, 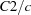

*a* = 14.4115 (8) Å
*b* = 6.2128 (3) Å
*c* = 27.5992 (15) Å
β = 104.379 (2)°
*V* = 2393.7 (2) Å^3^
*Z* = 8
Mo *K*α radiation
μ = 3.43 mm^−1^
*T* = 296 K
0.34 × 0.25 × 0.23 mm

#### Data collection

Bruker Kappa APEXII CCD diffractometer
Absorption correction: multi-scan (*SADABS*; Bruker, 2005 ▶) *T*_min_ = 0.389, *T*_max_ = 0.506
9373 measured reflections
2415 independent reflections
1670 reflections with *I* > 2σ(*I*)
*R*_int_ = 0.029

#### Refinement

*R*[*F* ^2^ > 2σ(*F* ^2^)] = 0.029
*wR*(*F* ^2^) = 0.075
*S* = 1.02
2415 reflections
163 parameters
H-atom parameters constrained
Δρ_max_ = 0.30 e Å^−3^
Δρ_min_ = −0.34 e Å^−3^

### 

Data collection: *APEX2* (Bruker, 2007 ▶); cell refinement: *SAINT* (Bruker, 2007 ▶); data reduction: *SAINT*; program(s) used to solve structure: *SHELXS97* (Sheldrick, 2008 ▶); program(s) used to refine structure: *SHELXL97* (Sheldrick, 2008 ▶); molecular graphics: *ORTEP-3 for Windows* (Farrugia, 2012 ▶) and *PLATON* (Spek, 2009 ▶); software used to prepare material for publication: *WinGX* (Farrugia, 2012 ▶) and *PLATON*.

## Supplementary Material

Crystal structure: contains datablock(s) global, I. DOI: 10.1107/S1600536813027426/hb7147sup1.cif

Structure factors: contains datablock(s) I. DOI: 10.1107/S1600536813027426/hb7147Isup2.hkl

Supplementary material file. DOI: 10.1107/S1600536813027426/hb7147Isup3.cml

Additional supplementary materials:  crystallographic information; 3D view; checkCIF report

## Figures and Tables

**Table 1:** Hydrogen-bond geometry (Å, °)

*D*—H⋯*A*	*D*—H	H⋯*A*	*D*⋯*A*	*D*—H⋯*A*
N3—H3*A*⋯N2	0.86	2.24	2.646 (3)	109
C6—H6⋯O1^i^	0.93	2.26	3.150 (3)	160

Symmetry code: (i) 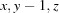.

**Fig. 1.**
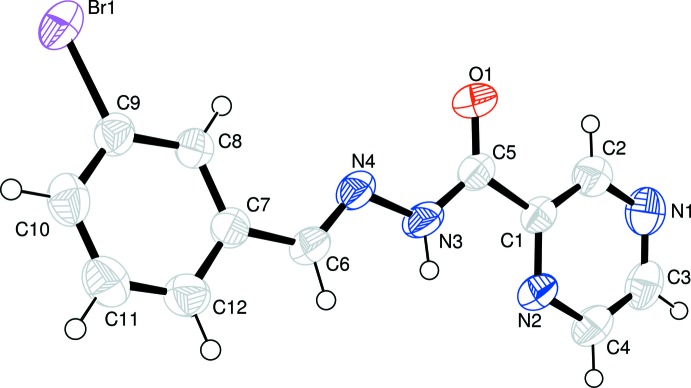
View of the title compound with displacement ellipsoids drawn at the 50% probability level.

**Fig. 2.**
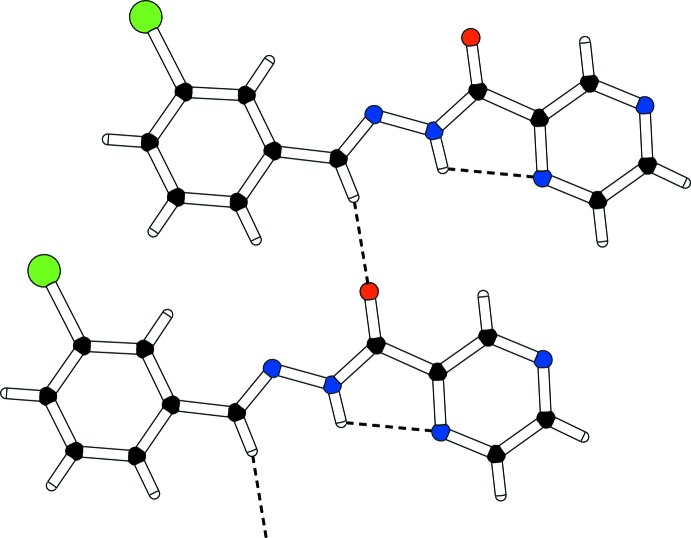
Packing diagram of the title compound, showing that molecules form polymeric chains.

## supplementary crystallographic information

### 1. Comment

The title compound (I), (Fig. 1) has been prepared in continuation of synthesizing different compounds containing pyrazine-2-carbohydrazide moiety (Hameed *et al.*, 2013*a*, 2013*b*).

In (I) the parts A (C1–C5/N1—N4/O1) and B (C6—C12/Br1) of pyrazine-2-carbohydrazide and 3-bromobenzaldehyde moieties are close to planar with r.m. s. deviations of 0.0259 Å and 0.0149 Å, respectively. The dihedral angle between A/B is 13.950 (54)°. There exist intramolecular H-bondings of N—H····N type (Table 1, Fig. 2) forming *S*(5) ring motif. Molecules are linked due to H-bonding of C—H····O type (Table 1, Fig. 2) forming C (6) chains. There exist π–π interactions at a distance of 3.9190 Å [*Cg*1—*Cg*2^i^ & *Cg*2—*Cg*1^i^: i = 1/2 - *x*, 1/2 - *y*, -*z*] and 3.9356 Å [*Cg*1—*Cg*2^ii^ & *Cg*2—*Cg*1^ii^: ii = 1 - *x*, 1 - *y*, -*z*], between the centroids of *Cg*1 (C1/C2/N1/C3/C4/N2) and *Cg*2 (C7—C12), respectively.

### 2. Experimental

The title compound was synthesized by the condensation of equimolar ratio of pyrazine-2-carbohydrazide with 3-bromobenzaldehyde, both dissolved in methanol. The resulting reaction mixture was stirred well and then refluxed for 5 h and allowed to cool over night. The precipitated solid was filtered, washed with petroleum ether and recrystallized from chloroform in pet ether and dried under reduced pressure over CaCl_2_ giving white crystalline compound. The crystals were re-grown in the same solvent system for crystallographic studies, yielding colourless prisms (m.p. 475–476 K).

### 3. Refinement

The H-atoms were positioned geometrically (N–H = 0.86 Å, C–H = 0.93 Å) and refined as riding with *U*_iso_(H) = *xU*_eq_(C, N), where *x* = 1.2 for all H-atoms.

### Crystal data

**Table:**

C_12_H_9_BrN_4_O	*F*(000) = 1216
*M**_r_* = 305.14	*D*_x_ = 1.693 Mg m^−^^3^
Monoclinic, *C*2/*c*	Mo *K*α radiation, λ = 0.71073 Å
*a* = 14.4115 (8) Å	Cell parameters from 1670 reflections
*b* = 6.2128 (3) Å	θ = 1.5–26.3°
*c* = 27.5992 (15) Å	µ = 3.43 mm^−^^1^
β = 104.379 (2)°	*T* = 296 K
*V* = 2393.7 (2) Å^3^	Prism, colorless
*Z* = 8	0.34 × 0.25 × 0.23 mm

### Data collection

**Table:**

Bruker Kappa APEXII CCD diffractometer	2415 independent reflections
Radiation source: fine-focus sealed tube	1670 reflections with *I* > 2σ(*I*)
Graphite monochromator	*R*_int_ = 0.029
Detector resolution: 8.00 pixels mm^-1^	θ_max_ = 26.3°, θ_min_ = 1.5°
ω scans	*h* = −17→17
Absorption correction: multi-scan (*SADABS*; Bruker, 2005)	*k* = −5→7
*T*_min_ = 0.389, *T*_max_ = 0.506	*l* = −34→34
9373 measured reflections	

### Refinement

**Table:**

Refinement on *F*^2^	Primary atom site location: structure-invariant direct methods
Least-squares matrix: full	Secondary atom site location: difference Fourier map
*R*[*F*^2^ > 2σ(*F*^2^)] = 0.029	Hydrogen site location: inferred from neighbouring sites
*wR*(*F*^2^) = 0.075	H-atom parameters constrained
*S* = 1.02	*w* = 1/[σ^2^(*F*_o_^2^) + (0.0348*P*)^2^ + 1.0725*P*] where *P* = (*F*_o_^2^ + 2*F*_c_^2^)/3
2415 reflections	(Δ/σ)_max_ = 0.001
163 parameters	Δρ_max_ = 0.30 e Å^−^^3^
0 restraints	Δρ_min_ = −0.34 e Å^−^^3^

### Special details

**Table:**

Geometry. All e.s.d.'s (except the e.s.d. in the dihedral angle between two l.s. planes) are estimated using the full covariance matrix. The cell e.s.d.'s are taken into account individually in the estimation of e.s.d.'s in distances, angles and torsion angles; correlations between e.s.d.'s in cell parameters are only used when they are defined by crystal symmetry. An approximate (isotropic) treatment of cell e.s.d.'s is used for estimating e.s.d.'s involving l.s. planes.
Refinement. Refinement of *F*^2^ against ALL reflections. The weighted *R*-factor *wR* and goodness of fit *S* are based on *F*^2^, conventional *R*-factors *R* are based on *F*, with *F* set to zero for negative *F*^2^. The threshold expression of *F*^2^ > σ(*F*^2^) is used only for calculating *R*-factors(gt) *etc*. and is not relevant to the choice of reflections for refinement. *R*-factors based on *F*^2^ are statistically about twice as large as those based on *F*, and *R*-factors based on ALL data will be even larger.

### Fractional atomic coordinates and isotropic or equivalent isotropic displacement parameters (Å^2^)

**Table:**

	*x*	*y*	*z*	*U*_iso_*/*U*_eq_	
Br1	0.62227 (2)	0.43755 (5)	0.23030 (2)	0.07124 (14)	
O1	0.34544 (12)	0.8178 (3)	−0.01354 (6)	0.0572 (5)	
N1	0.15137 (16)	0.8142 (4)	−0.15404 (8)	0.0650 (6)	
N2	0.23320 (14)	0.4402 (3)	−0.10560 (7)	0.0489 (5)	
N3	0.35102 (14)	0.4532 (3)	−0.01526 (7)	0.0501 (5)	
H3A	0.3313	0.3401	−0.0328	0.060*	
N4	0.41296 (14)	0.4300 (3)	0.03159 (7)	0.0470 (5)	
C1	0.25220 (16)	0.6348 (4)	−0.08500 (8)	0.0421 (5)	
C2	0.21160 (18)	0.8185 (5)	−0.10872 (9)	0.0565 (7)	
H2	0.2266	0.9502	−0.0927	0.068*	
C3	0.13278 (19)	0.6199 (5)	−0.17444 (10)	0.0620 (8)	
H3	0.0911	0.6087	−0.2060	0.074*	
C4	0.17254 (18)	0.4358 (5)	−0.15084 (9)	0.0567 (7)	
H4	0.1568	0.3041	−0.1668	0.068*	
C5	0.32089 (16)	0.6472 (4)	−0.03412 (8)	0.0432 (6)	
C6	0.43789 (17)	0.2363 (4)	0.04277 (8)	0.0488 (6)	
H6	0.4144	0.1287	0.0195	0.059*	
C7	0.50180 (16)	0.1759 (4)	0.09064 (8)	0.0429 (5)	
C8	0.52751 (15)	0.3190 (4)	0.13043 (8)	0.0441 (6)	
H8	0.5043	0.4593	0.1274	0.053*	
C9	0.58834 (16)	0.2486 (4)	0.17454 (8)	0.0457 (6)	
C10	0.62372 (18)	0.0415 (4)	0.17990 (10)	0.0557 (7)	
H10	0.6649	−0.0027	0.2098	0.067*	
C11	0.59711 (19)	−0.0986 (4)	0.14028 (11)	0.0599 (7)	
H11	0.6210	−0.2383	0.1434	0.072*	
C12	0.53564 (19)	−0.0348 (4)	0.09608 (10)	0.0538 (6)	
H12	0.5167	−0.1322	0.0699	0.065*	

### Atomic displacement parameters (Å^2^)

**Table:**

	*U*^11^	*U*^22^	*U*^33^	*U*^12^	*U*^13^	*U*^23^
Br1	0.0841 (2)	0.0729 (2)	0.04238 (16)	−0.00330 (16)	−0.01146 (13)	−0.00346 (13)
O1	0.0665 (11)	0.0511 (11)	0.0501 (10)	−0.0134 (9)	0.0073 (8)	−0.0144 (8)
N1	0.0630 (15)	0.0746 (17)	0.0523 (13)	0.0077 (13)	0.0045 (11)	0.0107 (12)
N2	0.0511 (11)	0.0532 (13)	0.0392 (10)	−0.0028 (11)	0.0048 (9)	−0.0096 (10)
N3	0.0553 (12)	0.0501 (13)	0.0367 (10)	−0.0022 (11)	−0.0042 (9)	−0.0109 (9)
N4	0.0491 (11)	0.0522 (13)	0.0345 (9)	−0.0025 (10)	0.0004 (8)	−0.0063 (9)
C1	0.0402 (13)	0.0502 (15)	0.0365 (11)	−0.0016 (11)	0.0110 (10)	−0.0043 (10)
C2	0.0604 (16)	0.0557 (17)	0.0519 (14)	0.0006 (14)	0.0110 (12)	−0.0003 (13)
C3	0.0508 (16)	0.089 (2)	0.0411 (13)	−0.0007 (15)	0.0011 (12)	0.0039 (14)
C4	0.0538 (15)	0.0694 (19)	0.0416 (13)	−0.0048 (14)	0.0018 (11)	−0.0103 (13)
C5	0.0432 (13)	0.0496 (15)	0.0375 (12)	−0.0041 (12)	0.0113 (10)	−0.0053 (11)
C6	0.0544 (15)	0.0505 (16)	0.0385 (12)	−0.0080 (13)	0.0058 (10)	−0.0083 (11)
C7	0.0425 (13)	0.0453 (15)	0.0408 (12)	−0.0035 (11)	0.0102 (10)	−0.0001 (10)
C8	0.0458 (13)	0.0422 (14)	0.0412 (12)	−0.0002 (11)	0.0048 (10)	0.0016 (11)
C9	0.0432 (13)	0.0499 (15)	0.0418 (12)	−0.0046 (12)	0.0064 (10)	0.0019 (11)
C10	0.0482 (14)	0.0611 (18)	0.0537 (15)	0.0025 (13)	0.0049 (11)	0.0138 (13)
C11	0.0620 (17)	0.0510 (17)	0.0680 (17)	0.0106 (13)	0.0183 (14)	0.0095 (13)
C12	0.0620 (16)	0.0472 (15)	0.0547 (15)	0.0002 (13)	0.0195 (13)	−0.0038 (12)

### Geometric parameters (Å, º)

**Table:**

Br1—C9	1.901 (2)	C3—H3	0.9300
O1—C5	1.213 (3)	C4—H4	0.9300
N1—C3	1.331 (4)	C6—C7	1.459 (3)
N1—C2	1.334 (3)	C6—H6	0.9300
N2—C4	1.335 (3)	C7—C8	1.390 (3)
N2—C1	1.335 (3)	C7—C12	1.392 (4)
N3—C5	1.341 (3)	C8—C9	1.383 (3)
N3—N4	1.384 (2)	C8—H8	0.9300
N3—H3A	0.8600	C9—C10	1.378 (4)
N4—C6	1.272 (3)	C10—C11	1.376 (4)
C1—C2	1.372 (3)	C10—H10	0.9300
C1—C5	1.506 (3)	C11—C12	1.376 (4)
C2—H2	0.9300	C11—H11	0.9300
C3—C4	1.369 (4)	C12—H12	0.9300
			
C3—N1—C2	115.5 (2)	N4—C6—C7	122.6 (2)
C4—N2—C1	115.7 (2)	N4—C6—H6	118.7
C5—N3—N4	121.85 (19)	C7—C6—H6	118.7
C5—N3—H3A	119.1	C8—C7—C12	119.9 (2)
N4—N3—H3A	119.1	C8—C7—C6	122.4 (2)
C6—N4—N3	113.77 (18)	C12—C7—C6	117.7 (2)
N2—C1—C2	122.1 (2)	C9—C8—C7	118.7 (2)
N2—C1—C5	117.5 (2)	C9—C8—H8	120.7
C2—C1—C5	120.4 (2)	C7—C8—H8	120.7
N1—C2—C1	122.1 (3)	C10—C9—C8	121.8 (2)
N1—C2—H2	118.9	C10—C9—Br1	118.40 (18)
C1—C2—H2	118.9	C8—C9—Br1	119.78 (19)
N1—C3—C4	122.7 (2)	C11—C10—C9	118.9 (2)
N1—C3—H3	118.7	C11—C10—H10	120.6
C4—C3—H3	118.7	C9—C10—H10	120.6
N2—C4—C3	121.8 (3)	C10—C11—C12	120.9 (2)
N2—C4—H4	119.1	C10—C11—H11	119.6
C3—C4—H4	119.1	C12—C11—H11	119.6
O1—C5—N3	125.1 (2)	C11—C12—C7	119.9 (2)
O1—C5—C1	121.9 (2)	C11—C12—H12	120.1
N3—C5—C1	113.0 (2)	C7—C12—H12	120.1
			
C5—N3—N4—C6	−176.9 (2)	C2—C1—C5—N3	177.4 (2)
C4—N2—C1—C2	0.3 (4)	N3—N4—C6—C7	−179.2 (2)
C4—N2—C1—C5	−179.6 (2)	N4—C6—C7—C8	10.8 (4)
C3—N1—C2—C1	0.5 (4)	N4—C6—C7—C12	−170.6 (2)
N2—C1—C2—N1	−0.7 (4)	C12—C7—C8—C9	1.3 (3)
C5—C1—C2—N1	179.2 (2)	C6—C7—C8—C9	179.9 (2)
C2—N1—C3—C4	−0.1 (4)	C7—C8—C9—C10	0.0 (4)
C1—N2—C4—C3	0.1 (4)	C7—C8—C9—Br1	−178.32 (17)
N1—C3—C4—N2	−0.3 (4)	C8—C9—C10—C11	−0.4 (4)
N4—N3—C5—O1	1.9 (4)	Br1—C9—C10—C11	177.9 (2)
N4—N3—C5—C1	−178.7 (2)	C9—C10—C11—C12	−0.5 (4)
N2—C1—C5—O1	176.8 (2)	C10—C11—C12—C7	1.8 (4)
C2—C1—C5—O1	−3.1 (4)	C8—C7—C12—C11	−2.3 (4)
N2—C1—C5—N3	−2.7 (3)	C6—C7—C12—C11	179.1 (2)

### Hydrogen-bond geometry (Å, º)

**Table:**

*D*—H···*A*	*D*—H	H···*A*	*D*···*A*	*D*—H···*A*
N3—H3*A*···N2	0.86	2.24	2.646 (3)	109
C6—H6···O1^i^	0.93	2.26	3.150 (3)	160

Symmetry code: (i) *x*, *y*−1, *z*.
